# A new mouse model of junctional epidermolysis bullosa: the *LAMB3* 628G>A knockin mouse

**DOI:** 10.1186/2194-7791-1-S1-A12

**Published:** 2014-09-11

**Authors:** Johanna Hammersen, Jin Hou, Stephanie Wünsche, Sven Brenner, Thomas Winkler, Holm Schneider

**Affiliations:** Department of Pediatrics and Adolescent Medicine, University Hospital Erlangen, Erlangen, Germany; Department of Biology, Nikolaus-Fiebiger-Center for Molecular Medicine, Friedrich-Alexander-University Erlangen-Nürnberg, Erlangen, Germany

Junctional epidermolysis bullosa (JEB) is a group of recessively inherited genodermatoses, characterized by tissue separation in the epidermal basement membrane due to defective anchoring proteins. The lethal Herlitz type of this disorder is caused by absence of laminin-332. Affected individuals suffer from widespread erosions of skin and mucous membranes and very often die within the first year of life. Mouse models lacking the α3- or γ2-chain of laminin-332 have been developed and a spontaneous ß3-knockout mouse exists, but all die shortly after birth. We generated a new mouse model of JEB by knockin of the point mutation 628G>A (p.E210K) in the gene encoding the laminin-332 ß3-chain, *LAMB3*. In compound heterozygous humans, this mutation has always been associated with lifelong skin blistering without reduced life expectancy.

Fourteen homozygous *LAMB3* 628G>A knockin mice were analyzed. Most of them showed skin blistering with tissue separation in the basement membrane soon after birth. Laminin-332 was almost completely absent. None of the homozygous *LAMB3* 628G>A knockin mice survived longer than 72 hours. *LAMB3* gene expression levels in heterozygous and homozygous *LAMB3* 628G>A knockin mice, however, were similar to wild-type mice. Analysis of the *LAMB3* transcript revealed alternative splicing in homozygous *LAMB3* 628G>A knockin mice: a 64 base-pair deletion of exon 7 led to a frame-shift and a premature termination codon. Due to alternative splicing, the phenotype of this new mouse model resembles that of knockout mice. The *LAMB3* 628G>A knockin mouse may contribute to a better understanding of the molecular basis of JEB.

